# Are thrombosis, progression, and survival in ET predictable?

**DOI:** 10.1038/s41408-024-01079-7

**Published:** 2024-06-25

**Authors:** Ghaith Abu-Zeinah, Katie Erdos, Neville Lee, Ahamed Lebbe, Imane Bouhali, Mohammed Khalid, Richard T. Silver, Joseph M. Scandura

**Affiliations:** 1 Richard T. Silver Myeloproliferative Neoplasms Center, Division of Hematology and Medical Oncology, Weill Cornell Medicine, New York, NY USA; 2 Weill Cornell Medicine-Qatar, Doha, Qatar

**Keywords:** Myeloproliferative disease, Risk factors

To the Editor,

Essential thrombocythemia (ET) is a chronic myeloproliferative neoplasm (MPN) that originates from a hematopoietic stem cell harboring a mutated *JAK2*, *CALR*, or *MPL* gene, or none of these three mutations (10–15% are “triple negative”). Although considered the most indolent MPN, ET is linked to burdensome vasomotor symptoms, and potentially fatal complications that include thrombosis, hemorrhage, and disease progression to myelofibrosis and aggressive myeloid neoplasms. Prognostic measures to identify those at greatest risk for thrombosis, progression, and death in ET (events) are important for timely risk-adapted intervention with available treatments, and for development of interventional trials to improve event-free survival (EFS). But predicting risks of events in ET has been difficult because ET is an uncommon and clinically heterogenous chronic disease. Predicting progression and excess mortality is even more challenging because these events typically occur decades after ET diagnosis [1]. Thus, retrospective analysis of large cohorts with sufficiently long follow-up is required to identify prognostic measures to stratify risk in patients with ET.

Prognostic models have been developed to assess the risk of thrombosis (IPSET-thrombosis [2]) or overall survival (OS) in ET (IPSET-survival [3], MIPSS-ET [4], and triple A [AAA] [5]). This journal recently published two large retrospective ET cohorts: Gangat et al. at the Mayo Clinic (Mayo) [6] and Loscocco et al. at the Florence Center Research and Innovation of Myeloproliferative Neoplasms (CRIMM) [7]. Both studies confirmed previously identified risk factors for thrombosis, progression and/or death in ET that include older age (Age ≥ 60), male sex, elevated white blood cell count (WBC > 11 × 10^9^/L), elevated absolute neutrophil count (ANC ≥ 8 × 10^9^/L), and low absolute lymphocyte count (ALC < 1.7 × 10^9^/L) at the time of presentation. We evaluated these parameters and current risk models in our cohort of 328 adult patients with ET treated at the Weill Cornell Medicine (WCM) Silver MPN Center over a median follow-up of 6 years [8]. This cohort was rigorously defined according to the 2022 World Health Organization diagnostic criteria and therefore all patients had a diagnostic bone marrow biopsy and had alternative diagnoses scrupulously ruled out. The methods of data collection, retrieval, and analysis used were previously described [9], and cohort characteristics are included in Supplementary Table 1.

**Fig. 1:**
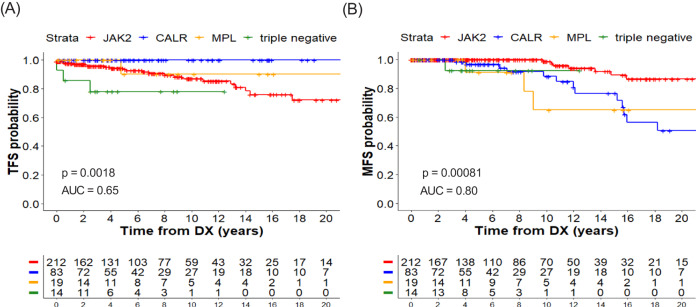
Thrombosis-free (TFS) and myelofibrosis-free survival (MFS) by driver mutation. **A** TFS of ET patients stratified by driver mutation. **B** MFS of ET patients stratified by driver mutation.

Thrombosis risk in both Mayo and CRIMM was related to age, mutated *JAK2*, and prior thrombosis, thereby affirming risk factors in the revised IPSET-thrombosis used to stratify patients into four tiers ranging from very low to high risk. Like Mayo and CRIMM, we found that patients with *JAK2*-mutated ET had significantly shortened thrombosis-free survival (TFS) compared to those with *CALR*-mutated ET (Fig. 1A). A total of 33 thromboses occurred (15 venous, 18 arterial) of which 27 (12 venous, 15 arterial) were experienced by *JAK2*-mutated patients. In multivariable models, the excess risk of thrombosis in *JAK2*-mutated ET compared to *CALR/MPL* was primarily due to venous thrombotic events (Supplementary Fig. 1). Similarly reported by CRIMM, we found IPSET-thrombosis mainly distinguished TFS between very-low and high-risk ET and poorly discriminated TFS between intermediate groups (Supplementary Fig. 2), with an overall underperformance as assessed by a receiver operator characteristic (ROC) area under the curve (AUC) of just 0.63. One issue affecting the performance of this, and similar prognostic models, may be the difficulty of predicting thrombotic events that can occur decades after the stratifying variables are assessed. The static nature of models like IPSET-thrombosis does not allow risk to change over time. For instance, therapy that is known to mitigate risk—such as blood pressure control, glycemic control, and use of antiplatelet, anticoagulant, and/or cytoreductive therapy—does not change risk stratification in these models. Mayo, CRIMM, and WCM all use cross-sectional data from initial presentation for analysis of IPSET-thrombosis and do not account for the effects of therapy and modifiable clinical factors such as blood counts. Nonetheless, Mayo and CRIMM showed that thrombosis risk was mitigated by aspirin [6] and cytoreductive therapy [7] establishing that true risk is dynamic.

Despite *CALR* mutations being associated with lower thrombosis risk, data from Mayo, CRIMM, and WCM point to significantly shorter myelofibrosis-free survival (MFS) in patients with *CALR* or *MPL* mutations compared to those with mutated *JAK2* (Fig. 1B) [6–8]. Progression to myelofibrosis occurred in 29 patients and was almost 12 times higher in our patients with mutated *CALR* and 16 times higher with mutated *MPL* compared to *JAK2*, as assessed by Cox multivariable analysis (MVA) that included age and sex (*CALR*/*JAK2*: HR 11.5, *p* = 0.008, *MPL*/*JAK2*: HR 16.4, *p* = 0.040). This is important because there are currently no validated risk models for predicting ET progression and these data indicate that future prognostic models for progression must incorporate driver mutation. Progression to MPN blast phase was an infrequent event (affecting 10 patients) and was not associated with driver mutation. Despite the differences in TFS and MFS, or because of them, there was no significant difference in OS between the ET driver genotypes (Supplementary Fig. 3). For this reason, it is not surprising that driver mutation is not a component of the mutation-enhanced prognostic model for OS, MIPSS-ET [4]. Across all three ET studies, there is clear evidence that separate risk models are needed for each of thrombosis, progression, and survival.

**Fig. 2:**
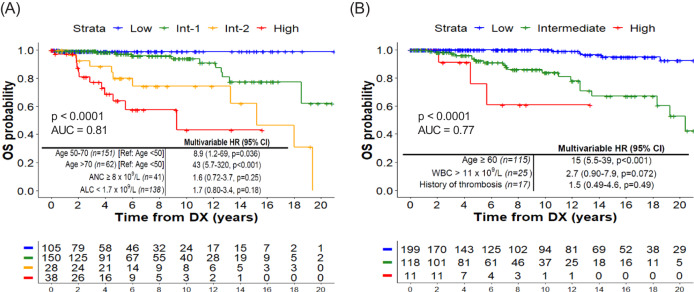
Overall survival (OS) models for ET patients. **A** OS of ET patients stratified by triple A risk. **B** OS of ET patients stratified by IPSET-survival risk.

Mayo and CRIMM developed and validated the AAA prognostic model for OS (stratified by Age, ANC and ALC) [5–7] and found that AAA outperformed IPSET-survival (stratified by Age, WBC, and previous thrombosis) [5]. In our WCM cohort, we found both AAA (Fig. 2A) and IPSET-survival (Fig. 2B) were prognostic for OS, with comparable performance as assessed by ROC (AUC of 0.81 vs 0.77, respectively, *p* = 0.18). Because age is always a risk factor for death and is always unmodifiable, the excess risk due to higher ANC and lower ALC, or higher neutrophil-to-lymphocyte (NLR) ratio [10], is of greater interest. Using Cox models, we found that ANC was independently associated with mortality (HR = 1.12, *p* = 0.003) when used as continuous variable but not as a binarized parameter at ≥8 × 10^9^/L (Supplementary Fig. 4). Binarized ANC was significant in univariable analysis of mortality when using an ROC-determined optimal cutoff of 6.9 (HR 2.5, *p* = 0.009), but was not significant in MVA with Age and ALC. Conversely, ALC was significant in univariable analysis and MVA of mortality when binarized according to ROC-determined optimal cutoff of 1.1 (Supplementary Fig. 5). When NLR was modeled instead with age, binarized at the ROC-determined optimal cutoff of 3.7, the model AUC was higher at 0.86 (Supplementary Fig. 5). To our knowledge, the biological link between high NLR and MPN-related complications has not been elucidated. We recently reported that *JAK2*^*V671F*^ impairs lymphoid differentiation of hematopoietic progenitors in vitro and in vivo across MPN subtypes [11]. The magnitude of lineage-biases in myeloid/lymphoid differentiation of *JAK2*^*V671F*^ HSPCs, or hematopoietic fitness, was highly correlated with EFS in our previously published prospective study of 107 patients with *JAK2*^*V671F*^ MPNs [12]. We, therefore, hypothesize that high ANC, low ALC, and high NLR are proxy measures of aggressive disease biology.

Our study of the WCM cohort agrees with key findings of the two large studies of patients with ET by Gangat et al. [6] and Loscocco et al. [7] and demonstrate that long-term risk of ET-related events can be stratified using readily available clinical information including age, driver mutations, and blood counts. Collectively, these independent studies firmly establish the following: (1) *JAK2*-mutated ET increases the risk of thrombosis compared to others with ET, and those with *CALR* mutant ET seldom experience thrombotic events; (2) *CALR* and *MPL* mutations significantly increase the risk of myelofibrosis progression; (3) driver mutation is not prognostic for OS; (4) the revised IPSET-thrombosis score accurately stratifies very-low and high long-term thrombotic risk but, overall, underperforms with real-world data; and (5) AAA, and to a lesser degree IPSET-survival, is prognostic for OS with age having the strongest influence on risk. In conclusion, these studies underscore the need for dynamic models for thrombosis, progression, and survival in ET that include modifiable factors which can be targeted to reduce risk.

The AAA is prognostic for OS when applied at the time of presentation but is not yet established for dynamic monitoring and predicting OS in ET. This model is appealing because it is tied to specific blood count abnormalities that could be linked to the underlying pathobiology of ET. However, in our data set, the prognostic value is dominated by the effect of age, which will always be tied to life expectancy and is a non-modifiable risk factor. Future models or revisions should consider de-emphasizing age to avoid undermining the higher relative mortality and morbidity of younger patients with ET, compared to their age-matched controls, than older patients (standardized mortality ratio analyses) [13, 14].

Addition of our WCM cohort with long-term follow-up to the recently published cohorts from Mayo and CRIMM provides high confidence in the risk factors identified, and the utility of available risk models for ET in long-term assessment of thrombosis, progression, and survival. However, these studies also support the need for accurate, dynamic, near-term predictive models. Near-term prediction of risk is a necessary step toward identifying cohorts of patients for targeted clinical trials to identify new risk-mitigating interventions. Increasing global use of electronic medical records provides access to large-scale, longitudinal datasets that can be used with health informatic resources for developing dynamic predictive models. Amidst the recent boom in artificial intelligence and machine learning, and the increasingly available resources for automated data retrieval, we need to collaboratively prove that life-threatening events in a chronic rare disease are indeed predictable and preventable.

### Supplementary information

Supplementary
