# Supplementary material for: Are thrombosis, progression, and survival in ET predictable?
Source: Blood Cancer J. 2024 Jun 25;14(1):103. doi: 10.1038/s41408-024-01079-7 (PMC11199579; doi:10.1038/s41408-024-01079-7)
Supplement: Supplementary file 1 — Supplementary [file 41408_2024_1079_MOESM1_ESM.pdf]

Supplementary Table 1. ET patient demographics by driver mutation

	Overall N = 328	JAK2 N = 212	CALR N = 83	MPL N = 19	Triple Negative N = 14	p-value
**Age at DX, yrs[1]**	53 [19-86]	53 [21-86]	52 [19-83]	52 [27-81]	62 [23-86]	0.3
**Sex[2]**						0.9
Female	213 (66)	140 (66)	52 (63)	12 (63)	9 (64)	
Male	115 (35)	72 (34)	31 (37)	7 (37)	5 (28)	
**Race/Ethnicity[2]**						0.9
White	230 (74)	147 (73)	62 (77)	11 (65)	10 (77)	
Black, Asian or Hispanic	71 (23)	47 (23)	16 (20)	5 (29)	3 (23)	
Other	11 (4)	7 (4)	3 (4)	1 (6)	0 (0)	
**IPSET-Thrombosis Risk at DX[2]**						<0.001
Very low	84 (26)	0 (0)	65 (78)	12 (63)	7 (50)	
Low	119 (36)	119 (56)	0 (0)	0 (0)	0 (0)	
Intermediate	30 (9)	0 (0)	18 (22)	5 (26)	7 (50)	
High	95 (29)	93 (44)	0 (0)	2 (11)	0 (0)	
**First line cytoreductive therapy[2]**						0.2
None	152 (46)	96 (45)	42 (51)	9 (47)	5 (36)	
Hydroxyurea	128 (39)	89 (42)	27 (33)	6 (32)	6 (43)	
Interferon	25 (8)	12 (6)	10 (12)	1 (5)	2 (14)	
Anagrelide	20 (6)	13 (6)	3 (4)	3 (16)	1 (7)	
Other	3 (1)	2 (1)	1 (1)	0 (0)	0 (0)	
**Follow up duration, yrs[1]**	6 [0-38]	7 [0-37]	6 [0-38]	7 [0-33]	4 [1-12]	0.7
**Lab values at DX[3]**						
Hematocrit (%)	41.0 [4.3]	42.0 [4.4]	39.3 [3.3]	39.4 [4.1]	37.5 [3.4]	<0.001
Hemoglobin (g/dL)	14.0 [3.4]	14.6 [3.9]	13.1 [1.2]	13.0 [1.5]	12.5 [1.1]	<0.001
Red blood cell count (10^6/uL)	4.6 [0.6]	4.8 [0.5]	4.4 [0.5]	4.5 [0.3]	4.4 [0.6]	<0.001
Mean corpuscular volume (fL)	88.8 [5.9]	88.4 [5.4]	90.7 [6.3]	87.5 [8.8]	86.5 [5.8]	0.5
White blood cell count (10^3/uL)	8.8 [6.4]	9.2 [7.8]	8.0 [2.8]	8.3 [5.2]	8.2 [4.2]	0.13
Platelets (10^3/uL)	764 [306]	688 [183]	891 [450]	830 [253]	1089 [414]	0.003
Lactate dehydrogenase (U/L)	224 [84]	214 [82]	248 [102]	246 [522]	245 [84]	0.3
**Outcomes[2]**						
Venous thrombosis	15 (5)	12 (6)	0 (0)	0 (0)	3 (21)	0.005
Arterial thrombosis	18 (6)	15 (7)	1 (1)	2 (11)	0 (0)	0.094
Progression to MF	29 (9)	9 (4)	16 (19)	3 (16)	1 (7)	<0.001
Progression to AML	10 (3)	9 (4)	2 (2)	2 (11)	0 (0)	0.3
Death	35 (11)	25 (12)	6 (7)	2 (11)	2 (14)	0.6

[1] Median [range]; [2] n (%); [3] Mean [SD]

Supplementary Figure 1. Thrombosis-free survival of ET patients stratified by driver mutation (A) Venous thrombosis-free survival. **(B)** Arterial thrombosis-free survival. **(C)** Uni- and multivariable analysis of venous thrombosis risk. **(D)** Uni- and multivariable analysis of arterial thrombosis risk.

(A)

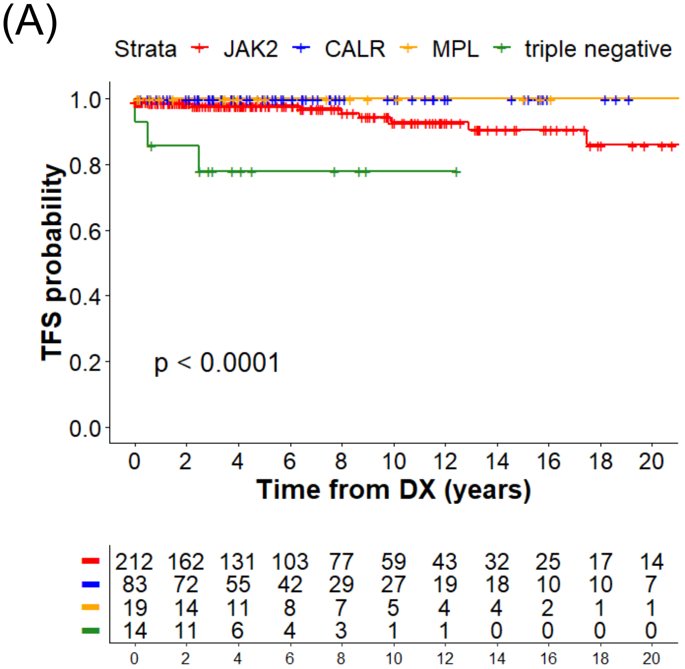

(B)

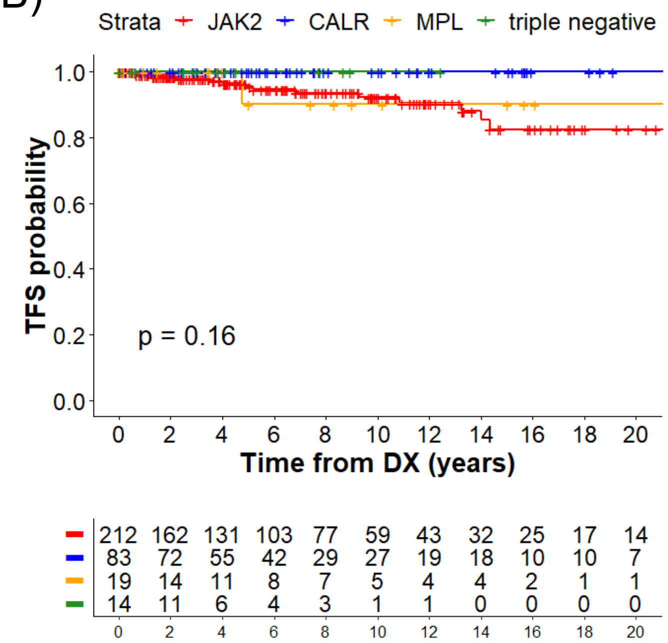

(C)

	**Univariable HR (95% CI, p-value)**	**Multivariable HR (95% CI)**
Age	1.0 (0.97-1.0, p=0.80)	0.99 (0.96-1.0, p=0.69)
Male sex (*n=115*)	0.51 (0.14-1.8, p=0.30)	0.52 (0.14-1.9, p=0.33)
CALR (*n=83*) [Ref: JAK2]	NA*	NA*
MPL (*n=19*) [Ref: JAK2]	NA*	NA*
Triple negative (*n=14*) [Ref: JAK2]	7.1 (1.9-27, p=0.004)	7.2 (1.8-28, p=0.005)

(D)

	**Univariable HR (95% CI, p-value)**	**Multivariable HR (95% CI)**
Age	1.0 (0.98-1.0, p=0.62)	1.0 (0.97-1.0, p=0.78)
Male sex (*n=115*)	1.1 (0.40-2.9, p=0.88)	1.2 (0.43-3.1, p=0.79)
CALR (*n=83*) [Ref: JAK2]	0.15 (0.02-1.2, p=0.068)	0.15 (0.02-1.2, p=0.070)
MPL (*n=19*) [Ref: JAK2]	1.2 (0.27-5.3, p=0.81)	1.2 (0.27-5.4, p=0.81)
Triple negative (*n=14*) [Ref: JAK2]	NA	NA

*HR not defined due to no events. Multivariable HR limits estimated using Monte Carlo methods:
CALR: avg HR: 0.10 (95% CI: 3.17e-09 - 0.44)
MPL: avg HR: 0.09 (95% CI: 3.2e-09 - 0.92)

Supplementary Figure 2. Thrombosis-free survival of ET patients stratified by revised IPSET-thrombosis risk

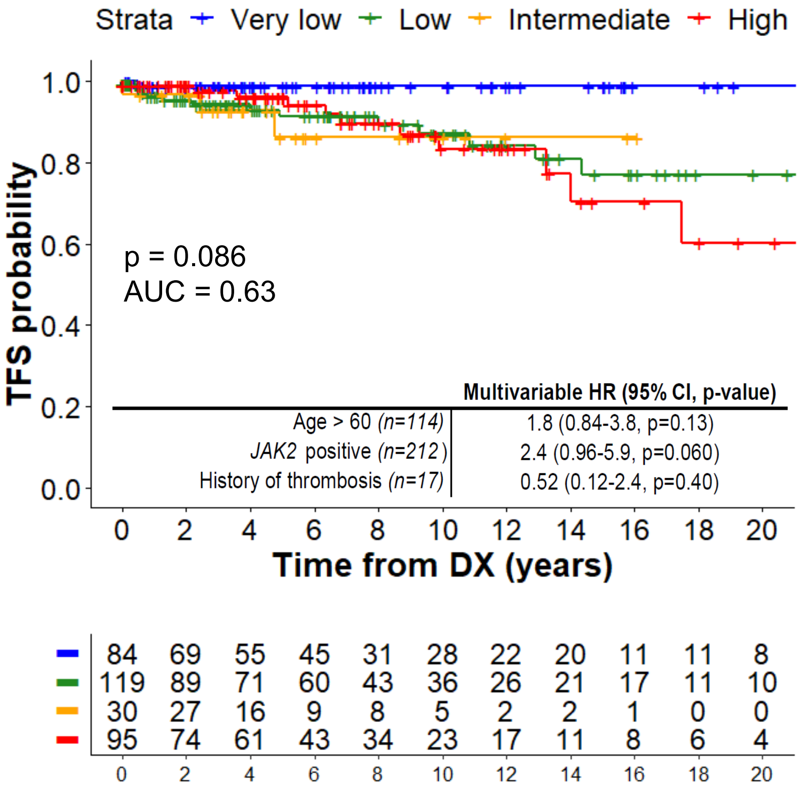

Supplementary Figure 3. Overall survival of ET patients stratified by driver mutation

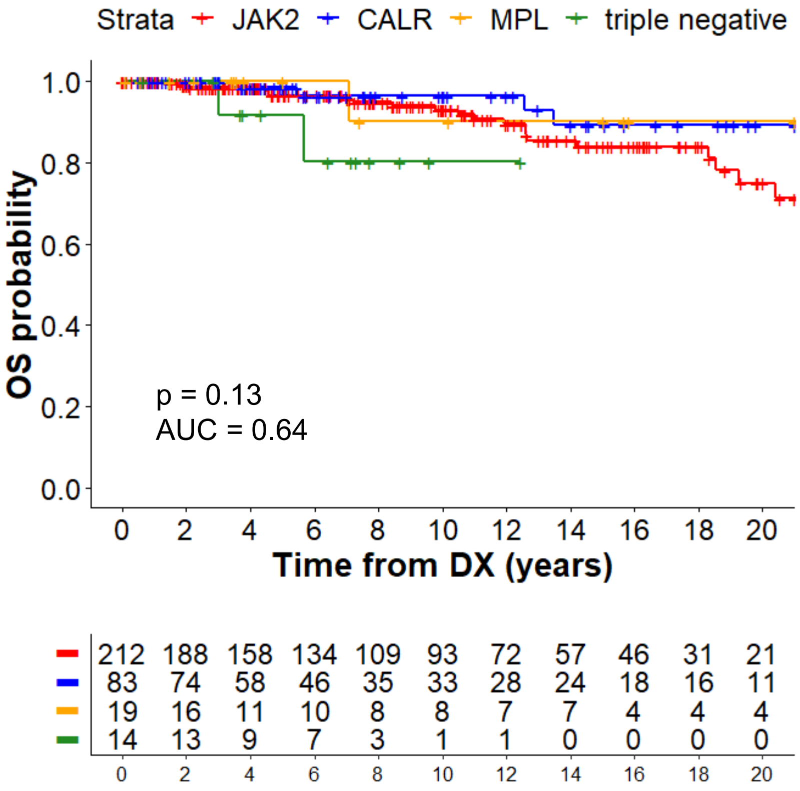

Supplementary Figure 4. Cox proportional hazard models of thrombosis (A) and mortality risk (B-D) **(A)** Univariable analysis of revised IPSET-thrombosis risk model variables. **(B)** Univariable analysis of IPSET-survival risk model variables. **(C)** Univariable analysis of Triple A risk model variables. **(D)** Uni- and multivariable analysis of Triple A risk model continuous variables.

(A)

	Univariable HR (95% CI, p-value)
Age > 60 *(n=114)*	2.1 (0.98-4.4, p=0.058)
JAK2 positive *(n=212)*	2.4 (1.0-5.9, p=0.049)
History of thrombosis *(n=17)*	0.66 (0.15-2.9, p=0.58)

(B)

	Univariable HR (95% CI, p-value)
Age ≥ 60 *(n=115)*	14 (5.4-38, p<0.001)
WBC > 11 x 10^9/L *(n=25)*	2.5 (0.88-7.3, p=0.084)
History of thrombosis *(n=17)*	1.3 (0.43-3.8, p=0.64)

(C)

	Univariable HR (95% CI, p-value)
Age 50-70 *(n=151)* [Ref: Age <50]	9.0 (1.2-69, p=0.035)
Age >70 (*n*=62) [Ref: Age <50]	41 (5.4-303 p<0.001)
ANC ≥ 8 x 10^9/L (*n*=41)	1.9 (0.86-4.3, p=0.11)
ALC < 1.7 x 10^9/L *(n=138)*	1.2 (0.63-2.4, p=0.54)

(D)

	Univariable HR (95% CI, p-value)	Multivariable HR (95% CI, p-value)
Age	1.1 (1.0-1.1, p<0.001)	1.1 (1.0-1.1, p<0.001)
ANC	1.1 (1.0-1.2, p=0.007)	1.1 (1.0-1.2, p=0.002)
ALC	1.1 (0.94-1.4, p=0.18)	1.1 (0.87-1.3, p=0.54)

Supplementary Figure 5. Overall survival of ET patients stratified by ALC, ANC, NLR (A) Overall survival stratified by ROC-determined optimal ALC threshold of 1.1. **(B)** Overall survival stratified by ROC-determined optimal ANC threshold of 6.9. **(C)** Overall survival stratified by ROC-determined optimal NLR threshold of 3.7. **(D)** Uni- and multivariable analysis of Triple A risk model variables, using the optimal ALC and ANC thresholds. **(E)** Uni- and multivariable analysis of mortality risk using Triple A risk model age groups and NLR threshold. **(F)** Uni- and multivariable analysis of mortality risk using age and NLR as continuous variables.

(A)

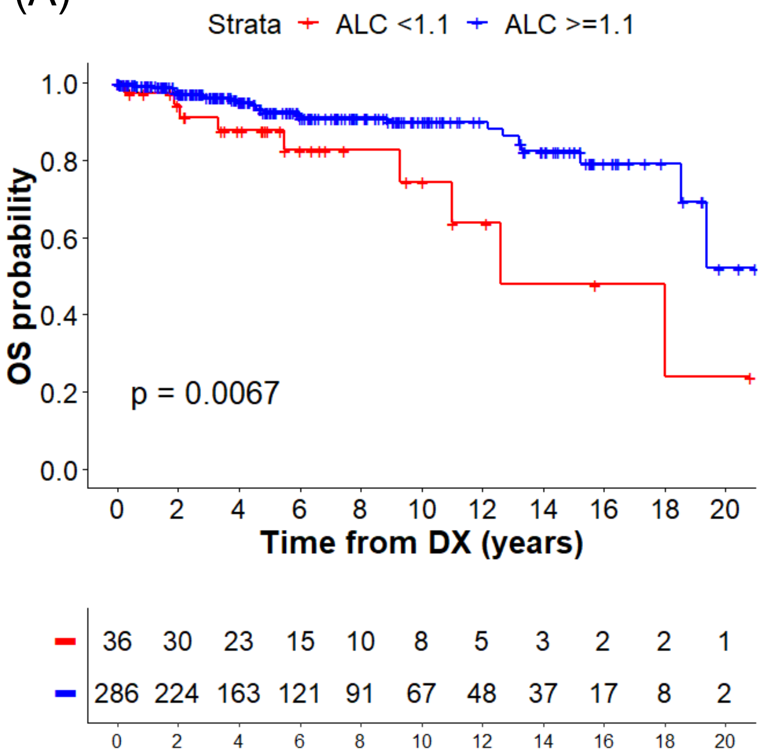

(B)

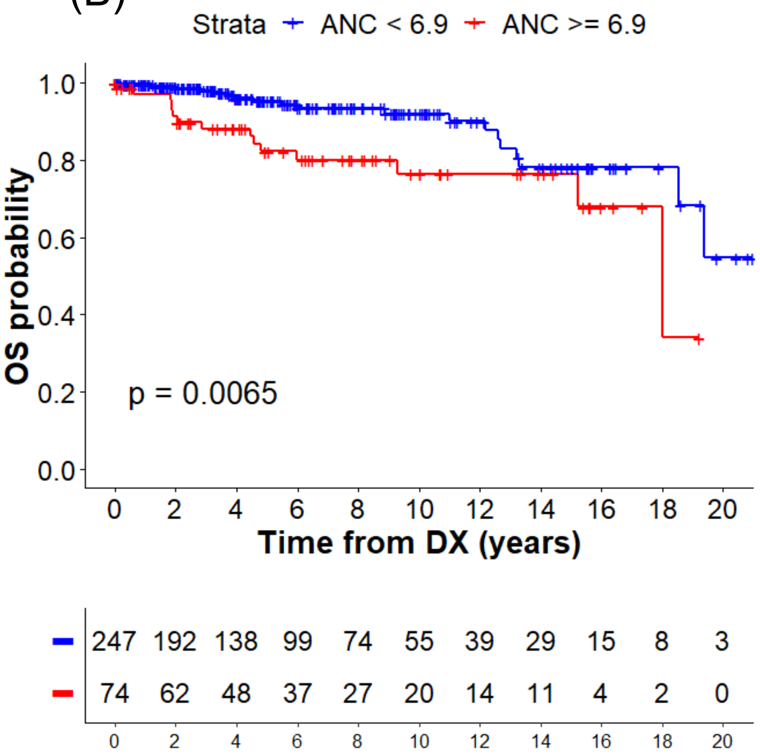

(C)

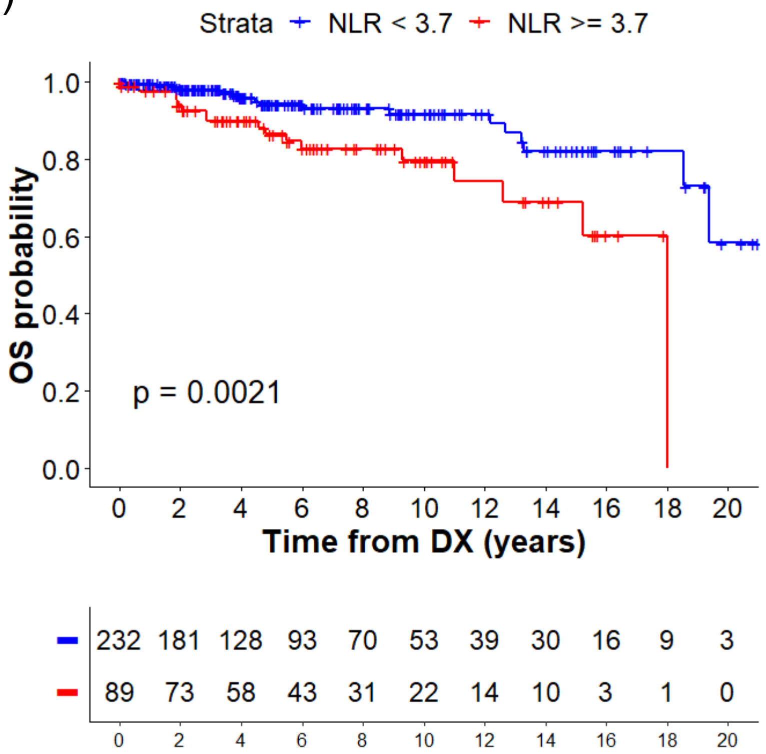

Supplementary Figure 5 cont. Overall survival of ET patients stratified by ALC, ANC, NLR (A) Overall survival stratified by ROC-determined optimal ALC threshold of 1.1. **(B)** Overall survival stratified by ROC-determined optimal ANC threshold of 6.9. **(C)** Overall survival stratified by ROC-determined optimal NLR threshold of 3.7. **(D)** Uni- and multivariable analysis of Triple A risk model variables, using the optimal ALC and ANC thresholds. **(E)** Uni- and multivariable analysis of mortality risk using Triple A risk model age groups and NLR threshold. **(F)** Uni- and multivariable analysis of mortality risk using age and NLR as continuous variables.

(D)

	Univariable HR (95% CI, p-value)	Multivariable HR (95% CI)
Age 50-70 *(n=151)* [Ref: Age <50]	9.0 (1.2-69, p=0.035)	8.4 (1.1-65, p=0.042)
Age >70 *(n=62)* [Ref: Age <50]	41 (5.4-303 p<0.001)	33 (4.4-250, p=0.001)
ANC ≥ 6.9 x 10^9/L *(n=74)*	2.5 (1.3-5.0, p=0.009)	1.7 (0.79-3.4, p=0.18)
ALC < 1.1 x 10^9/L *(n=36)*	2.8 (1.3-6.0, p=0.009)	2.2 (1.0-4.8, p=0.05)

(E)

	Univariable HR (95% CI, p-value)	Multivariable HR (95% CI)
Age 50-70 *(n=151)* [Ref: Age <50]	5.0 (1.1-22, p=0.034)	5.3 (1.2-24, p=0.029)
Age >70 *(n=62)* [Ref: Age <50]	22 (5.2-96 p<0.001)	20 (4.7-88, p<0.001)
NLR ≥ 3.7 *(n=89)*	2.9 (1.4-5.8, p=0.003)	2.3 (1.1-4.8, p=0.020)

(F)

	Univariable HR (95% CI, p-value)	Multivariable HR (95% CI)
Age	1.1 (1.1-1.1, p<0.001)	1.1 (1.1-1.1, p<0.001)
NLR	1.2 (1.1-1.3, p<0.001)	1.1 (1.0-1.2, p=0.024)
